# Pain relief by supraspinal gabapentin requires descending noradrenergic inhibitory controls

**DOI:** 10.1097/PR9.0000000000000659

**Published:** 2018-07-17

**Authors:** Dina L. Juarez-Salinas, Joao M. Braz, Katherine A. Hamel, Allan I. Basbaum

**Affiliations:** Department of Anatomy, University of California, San Francisco, San Francisco, CA, USA

**Keywords:** Gabapentin, Neuropathic pain, Noradrenergic, Conditioned place preference, Chemotherapy-induced neuropathic pain

## Abstract

**Introduction:**

Gabapentin regulates pain processing by direct action on primary afferent nociceptors and dorsal horn nociresponsive neurons. Through an action at supraspinal levels, gabapentin also engages descending noradrenergic inhibitory controls that indirectly regulate spinal cord pain processing. Although direct injection of gabapentin into the anterior cingulate cortex provides pain relief independent of descending inhibitory controls, it remains unclear whether that effect is representative of what occurs when gabapentin interacts at multiple brain loci, eg, after intracerebroventricular (i.c.v.) injection.

**Methods:**

We administered gabapentin i.c.v. in a mouse model of chemotherapy (paclitaxel)-induced neuropathic pain. To distinguish spinal from supraspinally processed features of the pain experience, we examined mechanical hypersensitivity and assessed relief of pain aversiveness using an analgesia-induced conditioned place preference paradigm.

**Results:**

Paclitaxel-treated mice showed a preference for a 100-μg i.c.v. gabapentin-paired chamber that was accompanied by reduced mechanical allodynia, indicative of concurrent engagement of descending controls. As expected, the same dose in uninjured mice did not induce place preference, demonstrating that gabapentin, unlike morphine, is not inherently rewarding. Furthermore, a lower dose of supraspinal gabapentin (30 μg), which did not reverse mechanical allodynia, did not induce conditioned place preference. Finally, concurrent injections of i.c.v. gabapentin (100 μg) and intrathecal yohimbine, an α2-receptor antagonist, blocked preference for the gabapentin-paired chamber.

**Conclusion:**

We conclude that pain relief, namely a reduction of pain aversiveness, induced by supraspinal gabapentin administered by an i.c.v. route is secondary to its activation of descending noradrenergic inhibitory controls that block transmission of the “pain” message from the spinal cord to the brain.

## 1. Introduction

Gabapentin (GP) is the first-line therapy for a variety of clinical neuropathic pain conditions.^11^ In preclinical settings, systemic GP is the most effective in nerve injury models where there is an upregulation of its target, the α2δ-1 subunit of voltage-gated calcium channels (VGCCs), notably in sensory neurons and in the spinal cord dorsal horn.^19,20^ There is also evidence for a supraspinal action of GP. For example, in a model of partial peripheral nerve injury, inhibition of spinal α2-adrenoreceptors counteracted the antiallodynia (mechanical hypersensitivity) produced by supraspinal GP.^31^ Furthermore, depletion of monoamines from descending projections by spinal administration of 6-hydroxydopamine, before a peripheral nerve injury, greatly reduced the antiallodynic effects of supraspinal GP.^30,31^ Finally, both supraspinal GP, in rodents, and systemic GP, in patients, significantly increase spinal cord tissue and cerebrospinal fluid levels of noradrenaline and its metabolites, respectively.^15,30^ Based on slice recordings, Takasu et al. suggested that the GP-induced descending controls originate in the locus ceruleus.^29^

These studies demonstrate that GP exerts its antiallodynic effects by an action at both supraspinal and spinal levels. Of course, the experience of pain also has a critical, cortically mediated aversive component that can be regulated by GP, and this presumably results from a supraspinal gabapentinoid action. In fact, in a recent report, Bannister et al.^2^ examined the effect of direct injection of GP into the anterior cingulate cortex (ACC), which is implicated in the aversive component of the pain experience. Using a conditioned place preference (CPP) model to monitor ongoing pain, they demonstrated a pain-relieving effect without a concurrent blockade of mechanical allodynia. The authors concluded that GP injection in the ACC does not engage descending inhibitory controls. Consistent with that conclusion, their parallel electrophysiological studies found no effect of ACC GP on noxious stimulus-evoked firing of spinal cord wide-dynamic-range neurons. On the other hand, they did record a reduced C-fiber-evoked activity, which suggests that descending controls are engaged, but perhaps not sufficiently to reverse mechanical allodynia.

Unclear is whether the action of GP in the ACC is representative of what occurs when GP interacts more generally at supraspinal sites, as for example, would occur after systemic injection. Ideally, this question could be addressed by injecting a compound into the ACC that counteracts the action of GP at the α2δ-1 subunit and studying its effects on the action of systemic GP. As this is, to date, not possible, here we asked whether intracerebroventricular (i.c.v.) injection of GP, which distributes the drug widely in the brain, can also regulate aversiveness of the pain experience, independently of its engagement of descending inhibitory controls. In our studies, we administered supraspinal GP (i.c.v.) immediately after a spinal cord (intrathecal [i.t.]) injection of the α2 noradrenergic antagonist, yohimbine, thereby blocking the effects of descending noradrenergic controls and asked whether GP was still pain relieving. We demonstrate that GP is not rewarding in the absence of injury, and that the pain relief produced by i.c.v. GP in the spared nerve injury (SNI) model of neuropathic pain, in fact, requires engagement of descending noradrenergic inhibitory controls.

## 2. Experimental procedures

### 2.1. Animals

All experiments were reviewed and approved by the Institutional Care and Animal Use Committee at the University of California, San Francisco. All animals were C57BL6 male mice between 6 and 8 weeks of age (22–30 g) and were randomized to treatment groups.

### 2.2. Neuropathic pain models

#### 2.2.1. Spared nerve injury

To produce mechanical hypersensitivity, we used the SNI model of neuropathic pain.^25^ The mice were anesthetized with 2% isoflurane, and then, we made a small incision on the left thigh, which exposed the sciatic nerve proximal to its trifurcation. Using 8-0 silk suture (Ethicon, Summerville, NJ), a tight ligature was tied around the common peroneal and sural nerve branches of the sciatic nerve, followed by their transection and removal of a 1.0-mm segment distal to the ligature. This procedure spares the tibial nerve. Overlying muscle and skin layers were closed separately with 6-0 silk and staples (Harvard Apparatus, Holliston, MA), respectively.

#### 2.2.2. Paclitaxel model of neuropathic pain

To produce mechanical hypersensitivity in a model that mimics a chemotherapy-induced neuropathic pain condition,^7,27^ we injected adult wild-type mice with 1.0 mg/kg of paclitaxel (Sigma, St. Louis, MO), dissolved in 40% dimethyl sulfoxide saline. The paclitaxel injections were repeated 4 times, every other day.

#### 2.2.3. Intracerebroventricular cannulation

Mice were deeply anesthetized with 2% isoflurane and placed in a stereotaxic instrument (Model 1900 Kopf). Scalp hair was removed with Nair; the scalp cleaned with Betadine and ethanol, after which a midline skin incision was made. A burr hole was made for stereotaxic targeting of the left lateral ventricle: −0.875, −0.3, and −2.7 mm. Next, we implanted a cannula (26 GA; Plastics One Inc, SW Roanoke, VA) that was secured to the skull with super glue and dental cement. The cannula was capped when access was not required. Mice were housed together after surgery.

#### 2.2.4. Angiotensin II test

To confirm proper placement of the cannula, we injected angiotensin II (ATII, 0.1 µg/µL in saline) using tubing connected to the needle of a 10-µL Hamilton syringe (33 GA; Plastics One Inc).^4^ The injection system was first filled with mineral oil, and then, 5.0 µL of ATII was front-loaded into the tubing. After manually injecting 1.0 µL of ATII, the mouse was placed into an empty cage that contained only a Petri dish of water. One minute later, we measured the duration of the drinking bout, over the next 5 minutes. Animals that drank for at least 10 seconds were considered to have an on-target cannula implant.

#### 2.2.5. Conditioned place paradigm

We used a 3-chambered custom-designed apparatus (Tap Plastics). Each chamber had different visual (dots vs stripes), olfactory (lemon vs vanilla extract), and flooring (smooth vs rough) cues, which distinguished the 2 end chambers. The neutral middle chamber had no cues to distinguish it. During habituation sessions, and on Pretest or Test days, the mice were always placed first into the neutral chamber.

On day 1 in the afternoon (3:30 pm–4:00 pm), the mice were habituated with full access to all chambers of the test apparatus. On day 2, the mice were habituated to the environment in the morning (9:00 am–9:30 am), and in the afternoon, their baseline preference for each chamber was recorded, for 30 minutes (“Pretest”). Importantly, in establishing whether there was a preference for each chamber, only the second 15 minutes of the video was scored. After Pretest readings were determined, a presumptive analgesic “pain reliever” (eg, GP) was paired with the less preferred chamber, and saline was paired with the more preferred chamber. On the conditioning days, days 3 and 4 (morning; 9:00 am), the control substance (saline) was administered (volume equivalent to that required for the test drug injection), and 45 minutes later, the mice were restricted for 30 minutes to 1 of the 2 chambers. In the afternoon (3:30 pm), we performed conditioning sessions for the GP. In this session, the mice were injected with GP and then restricted to the other chamber for 30 minutes. We injected GP either intraperitoneally (i.p.) 45 minutes before the mice were placed into the chamber or i.c.v. immediately before the animal was placed into the chamber. On day 5, the “Test” day, the mice were placed into the middle chamber and allowed to roam freely between the 3 chambers of the apparatus, after which their preference for each chamber during the second 15 minutes of the trial was recorded.

To calculate the CPP score, we subtracted the time (seconds) spent in each chamber of the box on the Pretest day from that of the Test day (CPP score = Test − Pretest). Conditioned place preference scores for each chamber (ie, drug-paired side or vehicle-paired side) for animals within each experimental group (ie, i.t. saline vs i.t. yohimbine; see below) were pooled. Within each group (ie, control vs paclitaxel-treated), CPP scores for the GP-paired chamber vs vehicle-paired chamber were analyzed with paired *t* tests.

#### 2.2.6. Mechanical threshold testing

For all groups, we recorded 3 days of baseline mechanical sensitivity before the peripheral nerve injury. Animals were habituated on a wire mesh for 2 hours, after which we used von Frey filaments (sizes 1.65, 2.44, 2.84, 3.22, 3.61, 3.84, 4.08, and 4.31) to measure mechanical withdrawal thresholds, using the up–down method.^8^ These filaments correspond to the following weights: 0.008, 0.004, 0.07, 0.16, 0.4, 0.6, 1, and 2 g, respectively. For each animal, we compared mechanical thresholds taken 1 week after nerve injury with the average of 3 presurgery baseline readings. If a particular animal's mechanical threshold was reduced by at least 50% relative to its average baseline, then it was considered to have nerve-injury–induced mechanical allodynia.

#### 2.2.7. Pharmacology

After we determined mechanical thresholds in the animals that underwent either the SNI or paclitaxel procedures, the cannula cap was removed and GP (or saline vehicle) was delivered (30 µg, 50 µg, or 100 µg/µL) using a 10-µL Hamilton syringe. After 10 seconds, the cap was replaced to ensure no fluid backflow. The investigator was blind to i.c.v. drug administered (saline vs GP).

For experiments that tested the effect of a spinally administered noradrenaline receptor antagonist on the ability of supraspinal GP to reverse mechanical allodynia, we injected yohimbine (5.0 μg/5.0 μL) into the lumbar i.t. space with a 30-gauge needle attached to a 10-μL Hamilton syringe. The yohimbine was injected immediately before the i.c.v. GP. The experimenter was blind to the i.t. drug injected (saline vs yohimbine).

#### 2.2.8. Statistical analysis

GraphPad Prism 6.0g software was used to analyze all data sets. All CPP data were analyzed using the Student paired *t* test of time spent in the saline-paired vs treatment-paired chamber within each group of mice (eg, we compared the preference of uninjured mice for the saline-paired chamber with the preference of uninjured mice for the GP-paired chamber). The dose-dependent antinociceptive effect of supraspinal GP (30, 50, and 100 μg) was analyzed using a 1-way analysis of variance (ANOVA) followed by the Tukey multiple comparison test (*df* = 4, F = 12.61). Mechanical thresholds before SNI, after SNI, and after i.c.v. GP + i.t. drug (saline or yohimbine) were compared using a 2-way repeated-measures ANOVA (RM-ANOVA) followed by the Sidak multiple comparison test. Comparisons of mechanical sensitivities for all 3 time points were made within each test group (ie, within the i.t. saline group or i.t. yohimbine group of animals [*df* = 14]). For all analyses, significance was set to be *P* < 0.05.

## 3. Results

### 3.1. Systemic gabapentin is pain relieving in paclitaxel-treated mice

In the first set of experiments, we used a chemotherapy-induced model of the neuropathic pain model, which is characterized by significant mechanical hypersensitivity of both forelimb and hind limb.^7,27^ We administered paclitaxel (1.0 mg/kg, i.p.) or vehicle (40% dimethyl sulfoxide, i.p.) every other day for 4 days. One week after the last injection, all animals were assayed for mechanical hypersensitivity, followed by a test of GP-induced CPP (GP-CPP). Animals that did not show at least a 50% drop in mechanical threshold relative to their baseline reading were not included in the study. Next, we assayed whether GP can also attenuate conscious aversiveness of the pain percept, ie, is “pain relieving.” To measure pain relief, we used a CPP paradigm in which mice learn to associate one chamber of the CPP test apparatus with an analgesic agent, in our studies, GP, and the other chamber with vehicle.^18^ If only the injured animals show a preference for the GP-paired chamber, then we concluded that the animals found GP to be a positive experience (ie, pain relieving), rather than a result of intrinsically rewarding properties of the GP.

**Figure 1.**
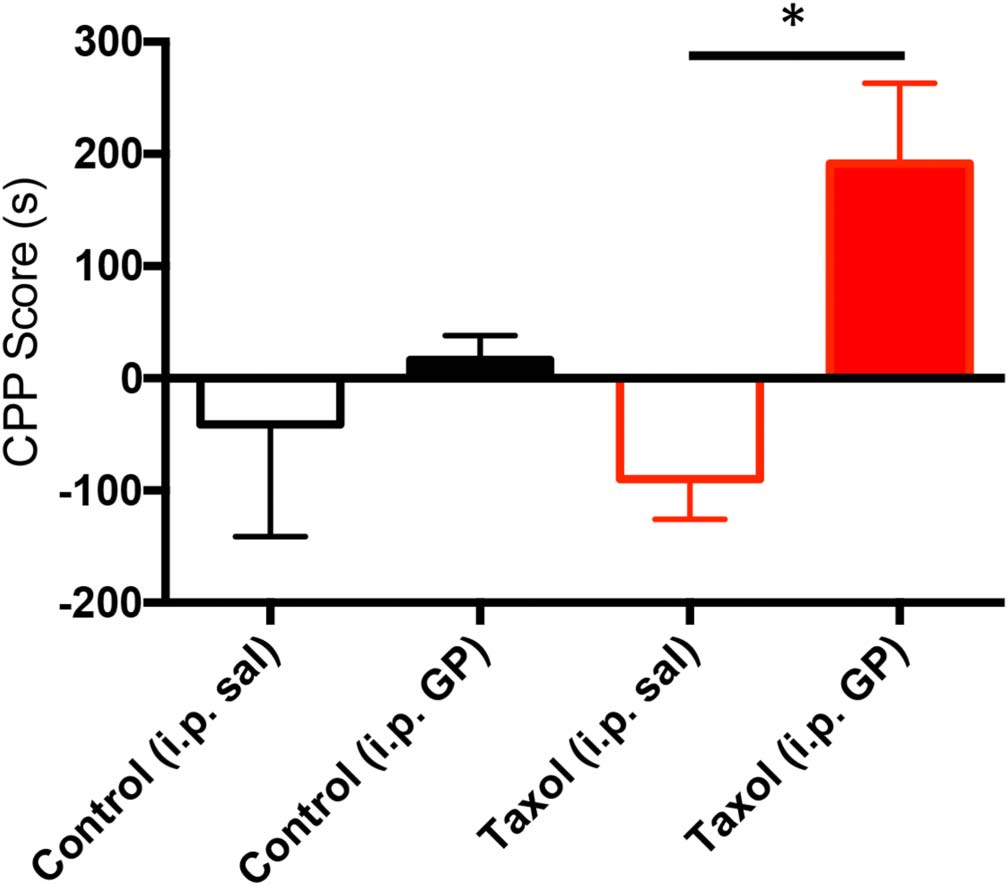
Systemic gabapentin (GP) is pain relieving in paclitaxel (Taxol)-treated mice. Histogram shows the CPP score in seconds for the saline-paired (open bars) and GP-paired (filled bars, 30 mg/kg i.p.) sides in uninjured (black bars, n = 5) and paclitaxel-treated mice (red bars, n = 6). Only the paclitaxel-treated mice show a preference for the GP-paired side (*P = 0.03; the Student *t* test compared with the saline-paired side). Data are mean ± SEM. CPP, conditioned place preference; i.p., intraperitoneal.

In these experiments, the mice received systemic GP (i.p. 30 mg/kg) 45 minutes before being restricted to one chamber of the CPP apparatus and received systemic saline when restricted to the other chamber. Figure 1 shows that paclitaxel-injected animals increased their preference for the GP-paired chamber (CPP score = 191.4 ± 71.73, the Student *t* test, *P* = 0.0294, N = 6), whereas the uninjured animals did not (CPP score = 16.47 ± 21.60, the Student *t* test, *P* = 0.64, N = 5). We conclude that systemic GP (30 mg/kg) is pain relieving; however, because mice without nerve injury showed no change in their preference for the GP-paired chamber, we conclude that GP is not inherently rewarding.

**Figure 2.**
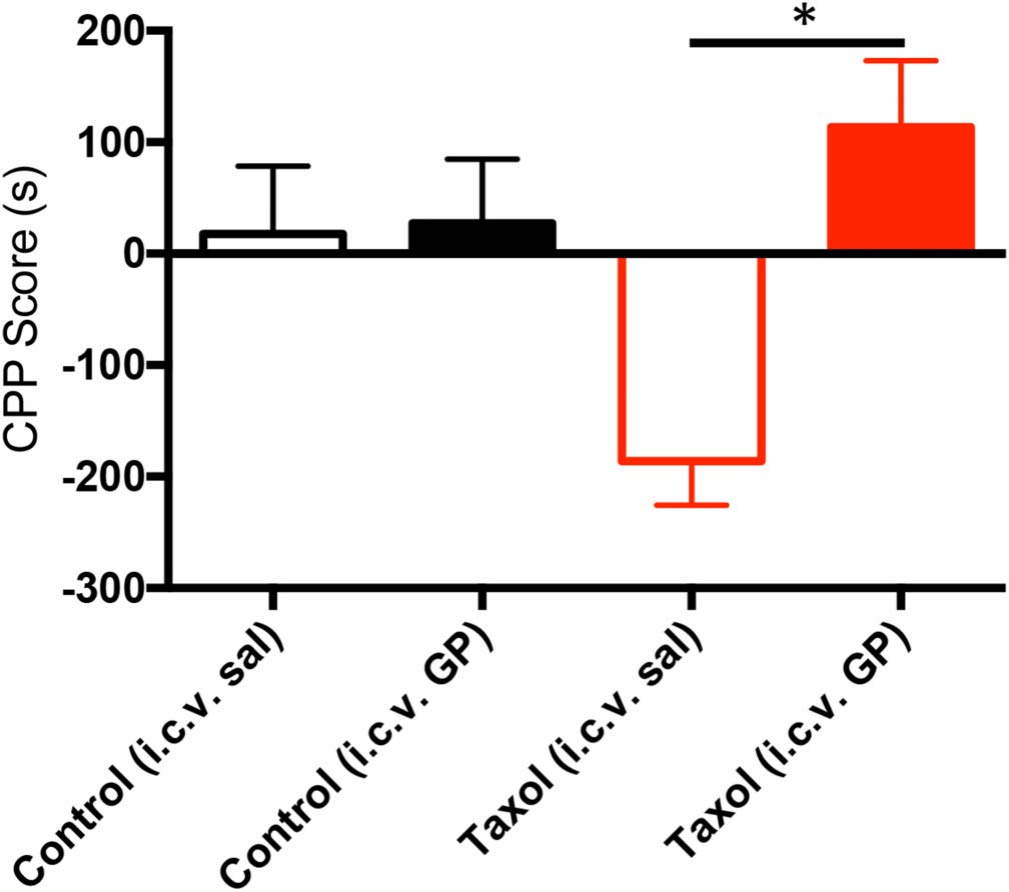
Supraspinal gabapentin (GP) is pain relieving in paclitaxel (Taxol)-treated mice. Histogram shows the CPP score in seconds for the i.c.v. saline-paired (open bars) and GP-paired (filled bars: 100 μg i.c.v.) sides in uninjured (black bars, n = 10) and paclitaxel-treated mice (red bars, n = 8). Only paclitaxel-treated mice show a preference for the i.c.v. GP-paired side (*P = 0.02; the Student *t* test compared with the saline-paired side). Data are mean ± SEM. CPP, conditioned place preference; i.c.v., intracerebroventricular.

Next, we examined the site of action of GP-mediated pain relief, focusing on a supraspinal mechanism. In these experiments, we implanted animals with a cannula that targeted the lateral ventricle. We studied 2 groups, one that received paclitaxel (N = 8) and another that received the vehicle (saline, N = 10). To assess accurate targeting of the ventricle, 6 days after cannula implant, the animals underwent an ATII-induced drinking test.^4^ Three days later, the mice in which ATII reliably induced drinking were tested in the CPP paradigm. Immediately after a lateral ventricle injection of GP (Fig. 2; 100 μg/1.0 μL, i.c.v.), the mice were placed into the chamber less preferred in the pretest trials. Again, i.c.v. GP induced a significant increase in preference for the GP-paired side of the apparatus, but this only occurred in paclitaxel-treated mice (CPP score = 113.5 s ± 59.49; N = 8, the Student *t* test, *P* = 0.0156). We conclude that supraspinal administration of GP is sufficient to relieve pain.

### 3.2. Supraspinal gabapentin administered at a dose that does not reverse mechanical allodynia does not relieve pain in paclitaxel-treated mice

As noted above, supraspinal GP, in the mouse, can reverse peripheral nerve injury–induced mechanical allodynia by engaging descending noradrenergic inhibitory controls.^28^ Here, we asked whether there is an i.c.v. dose of GP that is pain relieving, ie, induces place preference, but does not initiate descending controls, ie, does not normalize mechanical thresholds.

**Figure 3.**
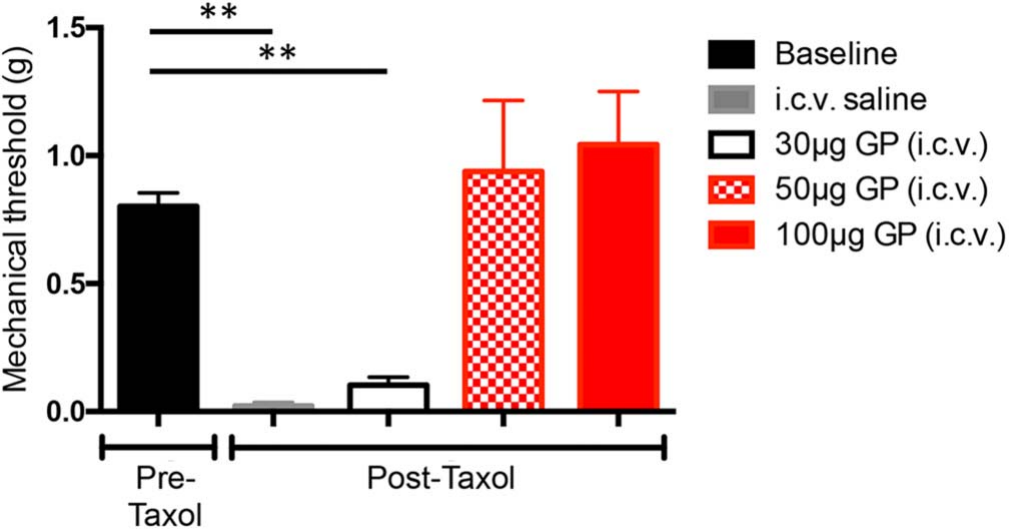
Dose–response for the antinociceptive effect of supraspinal gabapentin (GP) in paclitaxel (Taxol)-treated mice. Gabapentin was administered i.c.v. at 30 (n = 14), 50 (n = 8), or 100 μg (n = 9) to paclitaxel-treated mice. Both the 50 and 100 μg doses reversed mechanical allodynia of the hind paw; the low dose (30 μg) did not. Saline i.c.v. had no effect on mechanical thresholds (n = 8, 1-way ANOVA, the Tukey multiple comparisons test; *df* = 4; F = 12.61). Data are mean ± SEM. ANOVA, analysis of variance; i.c.v., intracerebroventricular. ** *P* < 0.01.

**Figure 4.**
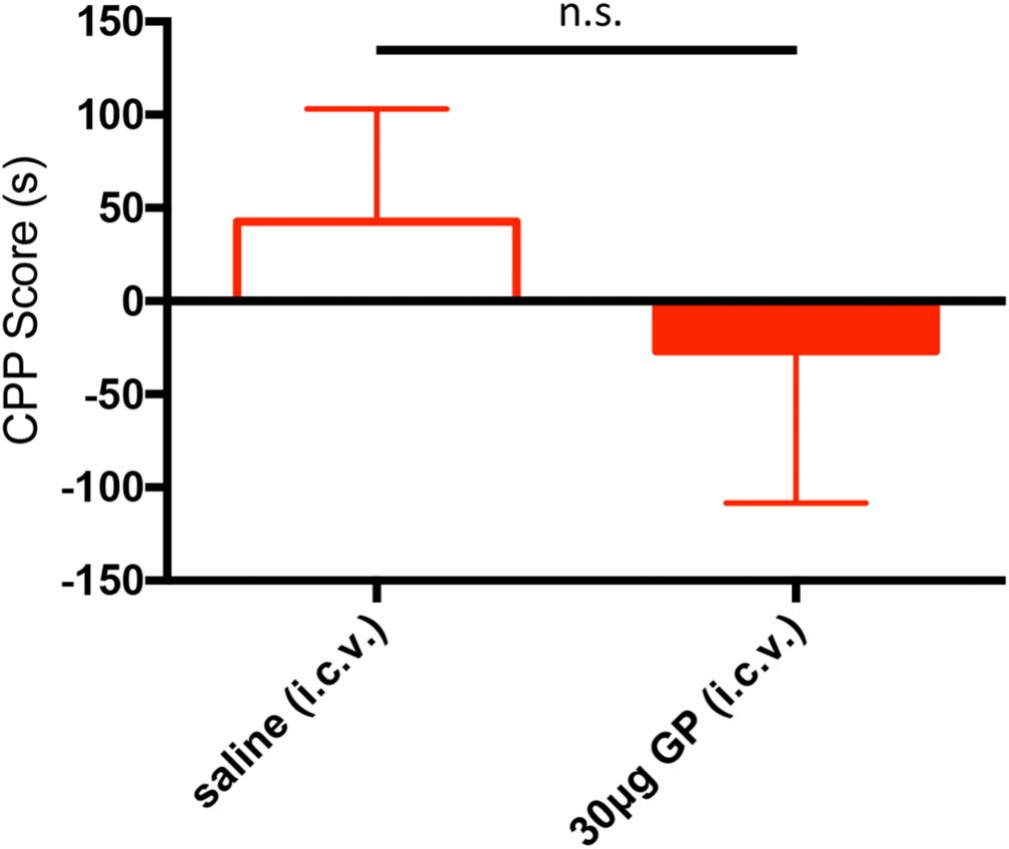
Low-dose supraspinal gabapentin (GP) is not pain relieving. Histogram shows the CPP score in seconds in paclitaxel-treated mice (n = 8) for the saline-paired (open red bar) and GP-paired (30 μg i.c.v., filled red bar) sides. There is no significant difference in CPP score between the 2 sides (the Student *t* test). Data are mean ± SEM. CPP, conditioned place preference; i.c.v., intracerebroventricular; n.s., not significant.

We first established a dose–response for the antinociceptive effect of i.c.v. GP on mechanical thresholds in the paclitaxel-treated mice (Fig. 3). Mechanical thresholds were assessed 1 week after the last paclitaxel injection. Next, the mice received 30 (N = 14), 50 (N = 8), or 100 μg/μL (N = 9) i.c.v. GP or vehicle (saline, N = 8). Figure 3 shows that i.c.v. GP, 50 and 100 μg, but not 30 μg, significantly reversed the paclitaxel-induced mechanical allodynia (0.937, 1.043, and 0.1034 g mean withdrawal thresholds of the injured paw, respectively; 1-way ANOVA, the Tukey multiple comparisons test; *df* = 4; F = 12.61). We conclude that the 30-μg dose is not sufficient to engage antinociceptive descending controls. Having established dose responsiveness, 1 week later we repeated the 30-μg i.c.v. dose in the GP-CPP paradigm, by pairing one chamber of the CPP apparatus with the GP. Figure 4 illustrates that these animals (N = 8) did not show a preference for the GP-paired side (Fig. 4; CPP score = −27.00 ± 81.49, the Student *t* test, *P* = 0.6281; *df* = 7). We conclude that 30-μg i.c.v. GP is below the threshold for relieving pain as measured in the CPP test. In other words, for i.c.v. GP to be pain relieving, it must be given at a dose that concurrently initiates descending controls to reduce mechanical allodynia.

### 3.3. Supraspinal gabapentin requires noradrenergic descending controls to relieve pain

We next asked whether the pain relief produced by i.c.v. GP persists after blocking descending noradrenergic inhibitory controls, which were previously implicated in the antiallodynic effect of supraspinal GP.^30^ Because the paclitaxel model of neuropathic pain induces whole-body hypersensitivity, we could not reduce completely an antiallodynic effect by i.t. (ie, lumbosacral) administration of yohimbine, an α2 adrenergic antagonist. For this reason, we used the unilateral SNI model of neuropathic pain^25^ in which the animal develops a prolonged mechanical hypersensitivity that is restricted to the injured hind paw. In these experiments, the mice received i.t. yohimbine (5.0 μg/5.0 μL) immediately before an antinociceptive i.c.v. GP injection (50 μg/1.0 μL).

**Figure 5.**
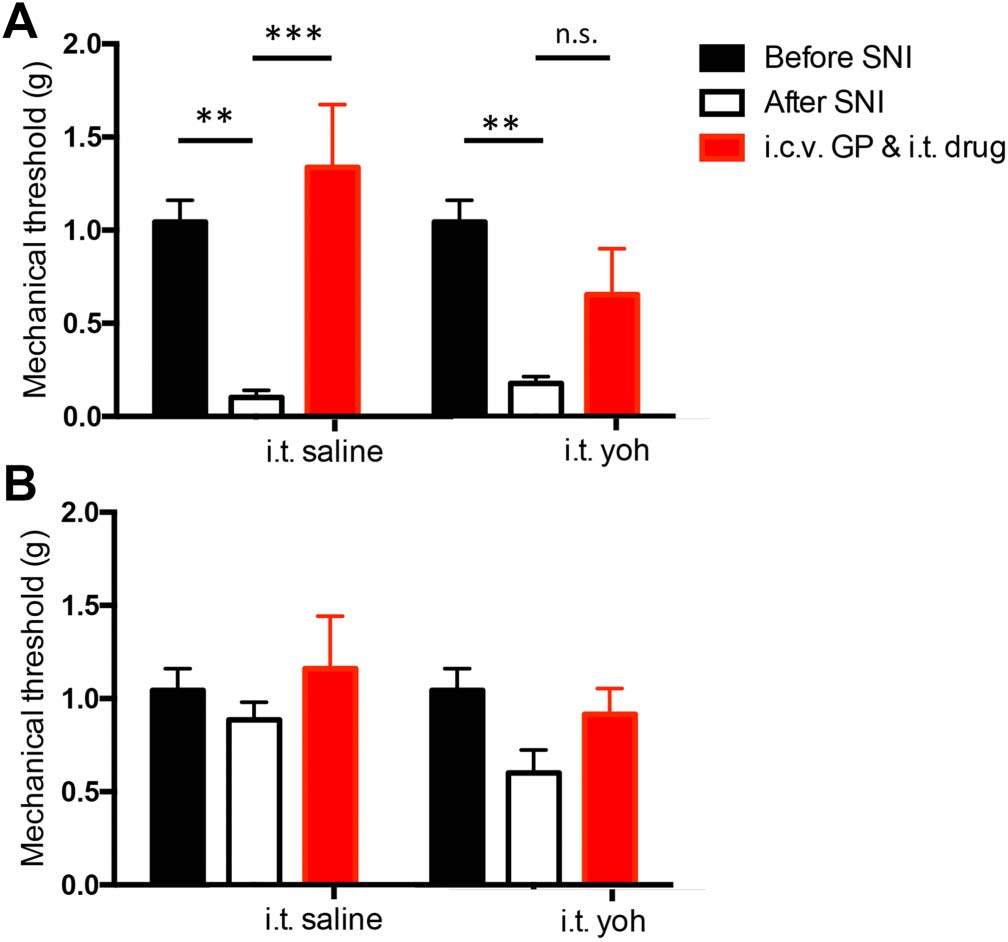
Blocking spinal α2 receptors reduces the antinociceptive effect of supraspinal gabapentin (GP). (A) After SNI, the mechanical withdrawal threshold of the injured paw differs significantly from its preinjury baseline level (**P < 0.01). Supraspinal GP (50 μg i.c.v.) increased the withdrawal threshold, demonstrating its antinociceptive effect. Intrathecal saline (n = 8) had no effect on the antinociceptive action of supraspinal GP (open black bars compared with filled red bars, ****P < 0.0001); i.t. yohimbine (5 μg, n = 8) reduced the antinociceptive effect of GP (open black bars compared with filled red bars, n.s., 2-way RM-ANOVA, the Sidak multiple comparison test; *df* = 14). (B) Supraspinal GP and i.t. yohimbine did not alter mechanical thresholds of the contralateral, uninjured paw. Data are mean ± SEM. ANOVA, analysis of variance; i.c.v., intracerebroventricular; i.t., intrathecal; n.s., not significant; RM-ANOVA, repeated-measures analysis of variance; SNI, spared nerve injury.

Using the von Frey test, we first compared the effect of yohimbine (N = 8) or vehicle (saline, N = 8) on the antiallodynic effect of 50-μg i.c.v. GP in animals that underwent SNI. Control animals received i.c.v. GP plus i.t. saline. Figure 5 shows that GP returned mechanical thresholds of the injured paw to preinjury, baseline levels (2-way RM-ANOVA, the Sidak multiple comparison test, *P* < 0.0001, when compared with post-SNI threshold; *df* = 14). By contrast, in animals that received i.t. yohimbine, mechanical thresholds did not change after GP (2-way RM-ANOVA, the Sidak multiple comparison test, not significant when compared with the post-SNI threshold). Importantly, neither i.c.v. GP nor combined i.c.v. GP and i.t. yohimbine altered the mechanical threshold of the uninjured paw (Fig. 5B; 2-way RM-ANOVA, the Sidak multiple comparison test, not significant), indicating that both the antinociceptive effect of GP, as well as the counteracting effect of i.t. yohimbine, are injury-dependent. In fact, in further control studies, we found that i.t. yohimbine, by itself, did not alter baseline mechanical thresholds in the absence (saline: 0.72 ± 0.07; yohimbine: 0.81 ± 0.03; N = 5) or presence of peripheral nerve injury (saline: 0.33 ± 0.04; yohimbine: 0.34 ± 0.03; N = 5). These findings parallel an earlier report that supraspinal GP does not engage descending inhibitory controls in the absence of injury, and that there is no tonic descending, yohimbine-sensitive, inhibitory control.^14,17,33^ Importantly, the latter studies only examined thermal withdrawal latencies. Our present results indicate that baseline mechanical sensitivity is also not under tonic noradrenergic control.

**Figure 6.**
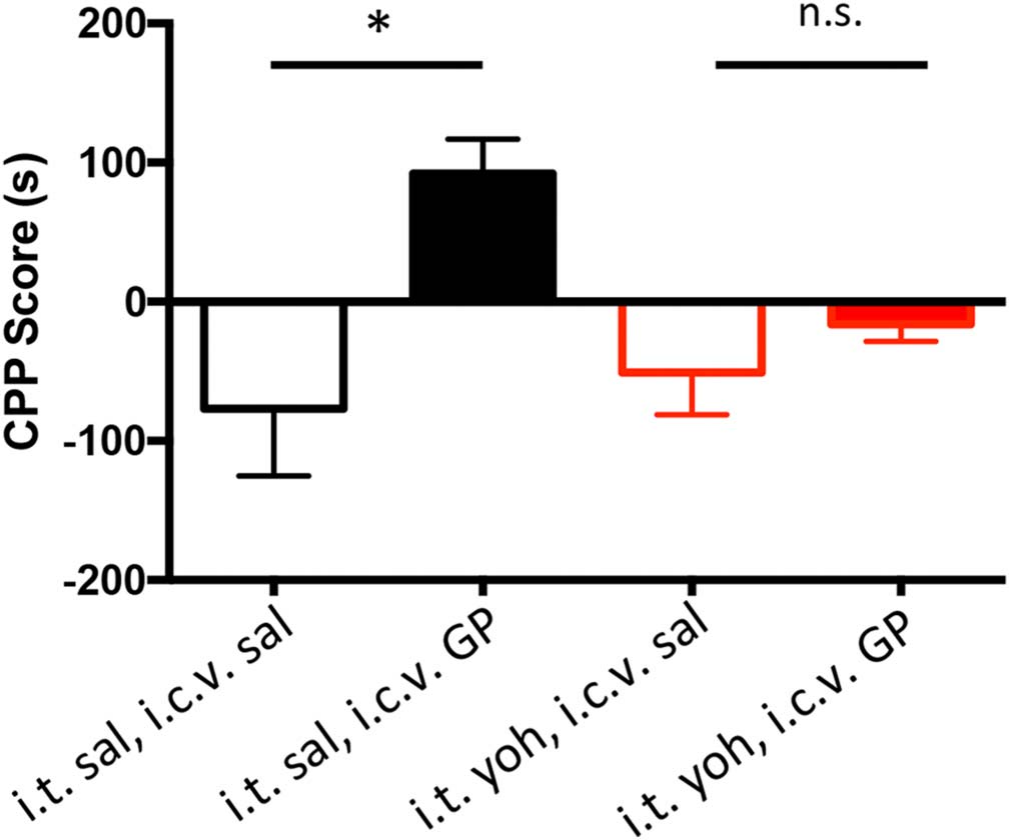
Supraspinal gabapentin (GP) requires noradrenergic descending controls to relieve pain. Conditioned place preference scores in seconds of mice for the i.c.v. saline-paired (open bars) and 100-μg i.c.v. GP-paired (filled bars) chambers: mice received i.t. saline (black bars, n = 8) or i.t. yohimbine (5.0 μg, red bars, n = 6). Mice that received i.t. saline show a preference for the GP-paired side (*P < 0.05; the Student *t* test compared with the saline-paired side). Yohimbine blocked the pain-relieving effect of supraspinal GP. Data are mean ± SEM. CPP, conditioned place preference; i.c.v., intracerebroventricular; i.t., intrathecal; n.s., not significant.

Next, we repeated the GP/yohimbine experiments, but here we used the CPP paradigm to monitor the pain-relieving effects of supraspinal GP. In these experiments, the mice received 100-μg i.c.v. GP and either i.t. yohimbine (5 μg/5 μL, N = 6; *df* = 5) or i.t. vehicle (saline, N = 8; *df* = 7) after which they were restricted to one chamber of the CPP apparatus. Figure 6 shows that mice that received i.t. saline paired with i.c.v. GP had a significant preference for the GP-paired chamber (Fig. 6; CPP score = 92.10 ± 24.76, the Student *t* test, *P* = 0.0360), a finding that is consistent with our earlier demonstration of the pain-relieving effects of supraspinal GP. By contrast, animals that received i.t. yohimbine paired with i.c.v. GP lost the preference for the GP-paired chamber (CPP score = −16.17 ± 12.41, the Student *t* test, *P* = 0.3927), ie, GP was no longer pain relieving. Collectively, these data indicate that the pain-relieving effect of i.c.v. GP is dependent on the engagement of a descending, noradrenergic antiallodynic inhibitory control system that operates at the level of the spinal cord. Based on these experiments, we conclude that GP, when administered by the i.c.v. route, does not exert a pain-relieving action at the level of the brain without concurrently engaging descending inhibitory controls.

## 4. Discussion

Preclinical studies have clearly demonstrated that supraspinal GP is not only antinociceptive, inhibiting withdrawal reflexes in response to an innocuous stimulus in an injury setting, but also relieves pain. Because pain relief cannot be measured by testing spinally mediated reflexes, here we evaluated pain relief using analgesia-induced CPP. In agreement with other reports that studied place preference in preclinical rodent models of neuropathic pain,^22,23^ we found that systemic GP is also pain relieving in a paclitaxel-induced chemotherapy model of neuropathic pain. On the other hand, we could not find an i.c.v. GP dose that induced pain relief without concurrent blockade of the paclitaxel-induced mechanical allodynia. In other words, an antinociceptive action at the level of the spinal cord is critical to the pain relief produced by i.c.v. GP. And consistent with other reports, we demonstrate that noradrenergic mechanisms mediate the descending inhibitory controls engaged by GP.

### 4.1. Systemic gabapentin–induced pain relief

How relevant are our findings to the mechanism of pain relief produced by systemic GP? Clearly, spinal, supraspinal, and even peripheral targets may be engaged and be relevant. Based on studies demonstrating that α2δ, the calcium channel subunit targeted by GP, determines abundance of VGCCs,^3,16^ it is hypothesized that GP presynaptically reduces the release of neurotransmitter from nociceptors through its regulation of the trafficking of α2δ-containing VGCCs to the membrane. Importantly, as peripheral nerve injury increases α2δ expression in sensory neurons and in the spinal cord^3,13,19^ and systemic GP profoundly inhibits the firing of dorsal horn nociresponsive neurons,^9,10^ it follows that a systemic action of GP can regulate pain processing by a direct action at the level of the spinal cord dorsal horn. Consistent with that hypothesis, i.t. GP is both antinociceptive and pain relieving in rodents.^2^

Somewhat surprisingly, it has proven difficult in patient populations to demonstrate consistently an antinociceptive action of gabapentinoids in tests of mechanical sensitivity, which is presumed to reflect an action at the spinal cord. In fact, there are very few randomized control trials that examined this question; some found an antiallodynic action of systemic GP,^1,5,6^ whereas other studies did not.^26^ Interestingly, in the latter study, the patients in whom pregabalin was effective (against pain of HIV neuropathy) were the most sensitive in a test of pin-prick hyperalgesia. Conceivably, in many studies that used quantitative sensory testing to assess the action of GP, the stimulus was not of sufficient intensity to detect an antinociceptive effect.

Also unexpectedly, despite the many reports of an antinociceptive action of GP after spinal injection, i.t. pregabalin failed to induce significant pain relief in a large clinical trial for neuropathic pain,^24^ despite the expression of α2δ in human dorsal root ganglion neurons.^12^ Importantly, as many of the subjects in that clinical trial had non-neuropathic low back or leg pain or mixed nociceptive and neuropathic back and leg pain, a trial directed only at patients with neuropathic pain may be necessary.

Based on the present findings and from results report by several other groups, it is very likely that systemic GP also exerts its antinociceptive and pain-relieving effects by an action in the brainstem. Through an hypothesized process of disinhibition that occurs in the locus ceruleus,^32^ GP would activate descending noradrenergic inhibitory controls at the level of the spinal cord. Consistent with that hypothesis, we found that i.t. yohimbine indeed reversed the pain-relieving effect of i.c.v. GP. As i.t. yohimbine did not alter mechanical thresholds, before or after nerve injury, we conclude that i.t. yohimbine, by itself, is not pronociceptive and not aversive. Furthermore, because the i.c.v. GP action required engagement of descending inhibitory controls, it was not surprising that we found a concurrent antinociceptive action and pain relief. When nociceptive signals originating in the spinal cord do not reach the brain, pain relief measured in the CPP test would inevitably be recorded. Conceivably, these supraspinally generated antinociceptive controls would summate and even potentially synergize with any peripheral or spinal action engaged by systemic GP.

Finally, recent studies of Bannister et al.^2^ reported that pain relief can be produced independently of the regulation of nociceptive processing at the level of the spinal cord. These authors demonstrated, in the rat, that microinjection of GP into the ACC induced a place preference in peripheral nerve–injured, but not in uninjured rats, without reduction of the mechanical allodynia or of the responsiveness of the dorsal horn wide-dynamic-range neurons.^2^ Those studies clearly demonstrated that the sensory and affective dimensions of the pain experience can be modulated independently. By contrast, separation of these 2 dimensions does not occur after i.c.v. GP, presumably because i.c.v. GP simultaneously engages multiple brain targets, including the ACC. The fact that systemic injection also engages many central nervous system loci, taken together with our finding that “pain relief” was blocked by spinal administration of an α2 antagonist, we suggest that descending inhibitory controls likely come into play when GP is administered systemically.

#### 4.1.1. Dopaminergic mechanisms in place preference

In a postsurgical pain model, Navratilova et al.^21^ recorded dopamine (DA) release in the nucleus accumbens concurrent with the pain relief produced by lidocaine-induced peripheral nerve block. The authors hypothesize that the DA release motivates the animal to seek pain relief (ie, exhibit a place preference for the lidocaine-paired chamber). In a more recent report, these authors found that the pain relief produced by intra-ACC GP is also associated with nucleus accumbens release of DA, and that withdrawal thresholds did not change.^2^ In light of the differential requirement of descending controls in those and the present studies, it follows that there are multiple modes through which GP can exert pain relief by an action in the brain. Based on the yohimbine antagonism of the pain relief produced by i.c.v. GP, an action at the locus ceruleus is likely involved. To what extent these multiple modes are engaged by a systemic injection of GP and perhaps most importantly, whether or not there is a target other than α2δ remains to be determined.

## Disclosures

The authors have no conflict of interest to declare.

This work was supported by NIH Grant R35 097306.
